# Adrenaline Auto‐Injector Prescribing in Primary Care in England: An Analysis of Non‐Standard Dosing

**DOI:** 10.1111/cea.70163

**Published:** 2025-11-02

**Authors:** Louise J. Michaelis, Thomas Owen, Andrew D. Bright, Lucy Sherwin‐Robson

**Affiliations:** ^1^ Newcastle Upon Tyne Hospitals NHS Foundation Trust Newcastle Upon Tyne UK; ^2^ Population Health Sciences Institute Newcastle University Newcastle Upon Tyne UK; ^3^ NHS Business Services Authority Newcastle Upon Tyne UK; ^4^ Gateshead Health NHS Foundation Trust, Queen Elizabeth Hospital Gateshead UK

**Keywords:** adrenaline, adult, allergy, anaphylaxis, autoinjector, paediatric, prescribing

## Abstract

**Introduction:**

The recommended first‐line treatment for anaphylaxis in the community is intramuscular injection of adrenaline. The treatment is a standardised dose of either a 150 μg or a 300 μg adrenaline auto‐injector (AAI) device depending upon the patient's weight. Currently, no standardised mechanisms exist to transition patients onto the higher 300 μg dose when they reach 25–30 kg (depending upon the manufacturer).

**Methods:**

We undertook analysis of NHS prescriptions data dispensed in the community in England to identify rates of non‐standard AAI dose prescribing. Non‐standard prescribing is defined as patients who are likely to have exceeded the 25–30 kg threshold but still received a 150 μg dose. Data were limited to the most recent AAI prescription for an individual patient that occurred in the last 2 years (December 2022–2024). Patient weight at the time of prescribing was approximated using an age‐to‐weight correlation model. AAI recommended switching weights, based on device metadata, were compared to the patients' approximated weight to identify non‐standard prescribing. Statistical comparison between rates of non‐standard prescribing and patient deprivation was computed using Kendall's Tau correlation coefficient. A complementary analysis to identify patients who received a 300 μg dose but were likely under the 25–30 kg threshold was also carried out.

**Results:**

Overall, 46,999 patients were identified as having received a 150 μg strength injector in their most recent AAI prescription; of these, over 95% received two or more devices in line with national guidance. Estimates based on age for weight growth centiles show that between 9480 (20.2%) and at least 1747 (3.7%) of those prescribed a 150 μg autoinjector were likely to exceed the weight threshold for this dose. Using a Resuscitation Council UK guideline of age 6 years for switching to a 300 μg dose, the estimated proportion prescribed a non‐standard AAI dose increases to 23,059 patients (49.1%). Estimated rates of non‐standard AAI prescribing were found to be higher in areas of England with the most deprivation. Conservative estimates found only 67 children likely under 25 kg and 330 children likely under 30 kg who received a 300 μg dose.

**Conclusions:**

This analysis of community AAI prescriptions in England suggests that underdosing of AAI prescriptions in children and adults is not uncommon. Healthcare professionals with patients at risk of anaphylaxis should review whether patients prescribed AAI devices have the appropriate device for their weight.

## Graphical abstract

Investigation into English primary care prescription data reveals that patients are receiving non‐standard doses of adrenaline auto‐injectors at all ages and across the whole of England. Guidance must be updated to include better dose‐switching procedures. At a minimum, patients aged 14 and older should be switched to 300 μg devices.

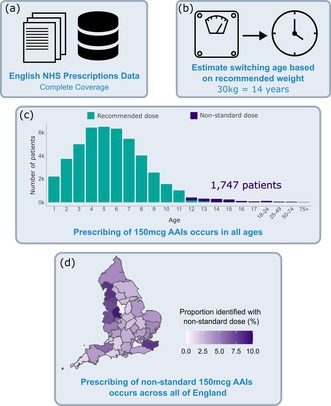

**Summary**

- We conducted a comprehensive review of primary care adrenaline autoinjector prescribing in England from 2022 to 2024.
- Prescribing of 150 μg autoinjectors for patients likely to weigh over 25‐30 kg was not uncommon.
- Professionals must review paediatric adrenaline autoinjector prescriptions, to align practice with national guidance.

## 1 Introduction

Anaphylaxis is defined as a serious potentially life‐threatening systemic hypersensitivity reaction that is usually rapid in onset [1, 2]. Up to 5% of the population is estimated to be at risk of food‐induced anaphylaxis [2], with 1 in every 300 people estimated to experience anaphylaxis at some point in their lives [3]. There are approximately 20 deaths reported each year due to this condition in the UK [4], although this may well be an underestimate [5].

The recommended first‐line treatment for anaphylaxis in the community is intramuscular injection of adrenaline [6]. Patients at risk of food and venom‐triggered anaphylaxis are often prescribed this in the form of adrenaline auto‐injectors (AAI), which are currently available in two different device dosages in the UK: 150 μg and 300 μg, with the latter dose recommended for individuals > 25–30 kg depending on the manufacturer. The RCUK recommends that children are switched to the stronger 300 μg dose AAI at age 6 [7]. In the UK, AAI prescriptions increased by 336% between 1998 and 2018, which represents a year‐on‐year increase of 11% [8, 9]. Annual increases in UK AAI prescribing have also been reported using national data over the span of almost two decades in a more recent study [10].

Delayed adrenaline injection is associated with poor outcomes and biphasic reactions [11, 12, 13, 14, 15], seen recently in a tragic example occurring in the North East of England [16]. Although recent data suggest that current AAIs have little impact on the rates of fatal anaphylaxis [10]. Treatment with sub‐optimal dosing of adrenaline during anaphylaxis may, among other risk factors, contribute to loss of life. The devastating story of a 13‐year‐old girl from East London exemplifies how additional external co‐factors can potentially contribute to a fatal outcome. During a national shortage of AAIs, she received a 150 μg device dose rather than the higher AAI dose recommended for her age/weight [17].

It is therefore imperative that all children or young people at risk of anaphylaxis should be prescribed a 300 μg AAI device when their weight surpasses the 25–30 kg mark. This is consistent with 3 major allergy reviews by the Royal College of Physicians [18], the House of Commons Health Committee [19] and the House of Lords Science and Technology Committee [20].

Our aim is to use data from NHS Prescriptions dispensed in the community in England to assess whether patients in receipt of an adrenaline auto‐injector (AAI) prescription are receiving a sufficient dose of Adrenaline.

## 2 Methods

### 2.1 Ethics Statement

NHS Business Services Authority (NHSBSA) is an arm's length body of the Department of Health and Social Care, collecting and using data in line with the exercise of its statutory and directed functions and responsibilities including those outlined in the NHS Act 2006 and, in relation to prescribing data, the Pharmaceutical and Local Pharmaceutical Services (Prescriptions, Payments and Listings) Directions 2013.

For this study, the NHSBSA undertook an analysis of pseudonymised NHS Prescriptions data in their secure data environment. The analysis of data is in accordance with the NHSBSA Privacy Notice. Patient information was analysed to identify and understand patterns and trends to help enhance NHS services and direct patient care. No data from Newcastle upon Tyne Hospitals NHS Foundation Trust was used in this analysis. Consultants at the trust have not had access to any patient identifiable data from the NHSBSA. For the purposes of clinical interpretation and discussion, they have only had access to the aggregated data that is contained within this manuscript.

### 2.2 Data Collection

NHSBSA processes NHS prescriptions dispensed within a primary care setting in England. The data are used to pay dispensing contractors and to provide prescribing and dispensing data to help NHS stakeholders track trends and to inform decisions.

Data were collected retrospectively by the NHSBSA from NHS prescription data for all English AAI prescriptions dispensed in primary care. The NHSBSA does not capture the expiry dates of dispensed AAI devices. However, to ensure prescriptions included in this analysis are still potentially valid, the data are limited to within a 2‐year window (December 2022–December 2024), corresponding to the maximum shelf life of an AAI as outlined by manufacturers. In the instances where a patient received multiple prescriptions containing an AAI within the timeframe, we only retain their most recent prescription for analysis. Patients were omitted from the analysis (< 0.1%) if there was no known age information held. The figures exclude around 1% of items which could not be linked to a patient between December 2022 and December 2024. For full details on data inclusion criteria see Appendix S1.

AAI metadata were extracted from the British National Formulary [21] and the Electronic Medicines Compendium [22]. The metadata collected include the recommended prescribing weight for an auto‐injector by brand and dosage (Table 1). Although recalled in May 2023, we include metadata for the 500 μg Emerade AAI because the time window selected in this analysis precedes the withdrawal date.

**TABLE 1:** Summary of auto‐injector metadata separated by brand and dosage strength.

BNF presentation	BNF presentation code	Minimum weight (kg)	Maximum weight (kg)
Emerade 150 μg/0.15 mL (1 in 1000) inj auto‐injectors	0304030C0BIAABF	15	30
EpiPen Jr. 150 μg/0.3 mL (1 in 2000) inj auto‐injectors	0304030C0BEAAA2	7.5	25
Jext 150 μg/0.15 mL (1 in 1000) inj auto‐injectors	0304030C0BHAABF	15	30
Emerade 300 μg/0.3 mL (1 in 1000) inj auto‐injectors	0304030C0BIABA3	30	N/A
EpiPen 300 μg/0.3 mL (1 in 1000) inj auto‐injectors	0304030C0BEABA3	25	N/A
Jext 300 μg/0.3 mL (1 in 1000) inj auto‐injectors	0304030C0BHABA3	30	N/A
Emerade 500 μg/0.5 mL (1 in 1000) inj auto‐injectors	0304030C0BIACBG	60	N/A

*Note:* Auto‐injector metadata were collected from the British National Formulary (BNF), Electronic Medicines Compendium and from the manufacturer's website. The British National Formulary presentation code was used to identify auto‐injector prescriptions.

#### 2.2.1 Using Age as a Proxy for Weight

Guidelines outlining switching from 150 μg to 300 μg AAI make recommendations based on the weight of an individual. The maximum allowable weight a patient can be is brand specific and is either 25 kg or 30 kg for the 150 μg AAI device. The NHSBSA does not capture patient weight data. Instead, the age of an individual, at the date of prescription, was used as a proxy for weight. The World Health Organisation (WHO) growth charts for UK children, obtained from the Royal College of Paediatrics and Child Health [23], were used to estimate the ages at which children surpass the switching weight thresholds of 25 kg and 30 kg as determined by the brand of the AAI injector. The 0.4th percentile was chosen for the analysis since 99.6% of patients are expected to exceed a given weight at a particular age. This means that we can be more confident that the patients included in the analysis are above the switching weight and in receipt of a non‐standard dose. For example, we expect 99.6% of patients to exceed 25 kg at age 12, and 99.6% of patients to exceed 30 kg at age 14. A drawback of the 0.4th percentile is that, although we can be more confident that the identified patients have a non‐standard dose, this is an underestimate of the true population of patients with non‐standard doses, as many patients will exceed the weight threshold much sooner. For this reason, we also present the results using the 50th percentile. For the 50th percentile, to surpass the 25 kg and 30 kg thresholds, patients need to be at least 8 and 10 years old, respectively. Therefore, depending on the percentile used (0.4th or 50th) and the weight threshold (25 kg or 30 kg based on the brand of the AAI dispensed), the identified switching age can vary from 8 years to 14 years. The results using the 25th and 75th percentiles are included in Appendix S1. The NHSBSA does not directly capture patient ethnicity; however, the RCPCH growth charts used to estimate a cut‐off age do consider the ethnic diversity of the UK [23]. This is key, given that a recent report by the BSACI registry concluded that there are inequalities across ethnic groups in the UK when it comes to anaphylaxis treatment [24]. A complete overview of estimated switching ages at 25 kg and 30 kg for different percentiles is provided in Table 2.

**TABLE 2:** Summary of the estimated age where patients exceed a weight.

Percentile	Age at 30 kg (Male)	Age at 30 kg (Female)	Age at 25 kg (Male)	Age at 25 kg (Female)
0.4	13.5	13.16	11.58	11.83
25	10.5	10.42	8.67	8.67
50	9.5	9.33	7.75	7.67
75	8.4	8.33	6.83	6.83

*Note:* The age males and females exceed 25 kg and 30 kg were estimated using growth charts available on the Royal College Paediatrics Child Health website. In this analysis we used the most conservative estimates (0.4th percentile). Estimated ages are provided for other percentiles for completeness.

The Resuscitation Council UK (RCUK) has published guidelines for the treatment of anaphylaxis [25]. Within the guidelines are recommendations that children younger than 6 years old should be prescribed 150 μg auto‐injectors, and older patients should be prescribed 300 μg AAIs. Results are also presented using this guidance to provide a switching age.

### 2.3 Statistical Analysis

By comparing the age of an individual, the brand of AAI dispensed, and the estimated switching age, we were able to categorise whether patients were prescribed the recommended dose of AAI.

#### 2.3.1 Geographic and Deprivation Reporting of Auto‐Injector Prescribing

For geographic reporting, each patient's postcode was mapped to an LSOA (2011 classification) based on the latest available mapping [26]. Within this report, Integrated Care System (ICS) classifications (April 2023) are then based on mappings from LSOAs [26]. There are 42 ICSs in England. They are local partnerships formed by NHS organisations and upper‐tier local councils to develop shared plans and joined‐up services.

For deprivation reporting, each LSOA (2011 classification) is aligned to an Index of Multiple Deprivation (IMD) decile (2019 classification) based on mappings published with the English indices of deprivation 2019 [27]. IMD decile classification combines scoring across seven deprivation indicators. IMD scoring is assigned at an LSOA level and can be used to assign a LSOA to an IMD decile or rank ICSs based on relative deprivation scores. The first IMD decile represents the 10% of LSOAs that are the most deprived in England.

To address population differences, results show the proportion of patients prescribed non‐standard 150 μg auto‐injectors relative to all patients with a 150 μg prescription.

#### 2.3.2 Statistical Comparison of Deprivation With Auto‐Injector Prescribing

The associations between IMD decile and the rates of patients with non‐standard AAI doses at an ICS level were quantified using a one‐tailed Kendall's tau correlation coefficient. This method was selected as IMD is ordinal, and the proportion of non‐standard AAI doses is continuous [28]. A negative association between deprivation levels and the proportion of patients with non‐standard AAI doses was hypothesised.

## 3 Results

### 3.1 Auto‐Injector Prescribing Rates Over Time

When the NHSBSA processes prescriptions, it is not always possible to capture the NHS number of the patient. The proportion of AAI prescription items that could be linked to individual patients was around 94% in 2015/16 and increases to around 99% in 2023/24. This means that data relating to auto‐injector prescribing represent most, but not all, patients.

**FIGURE 1:**
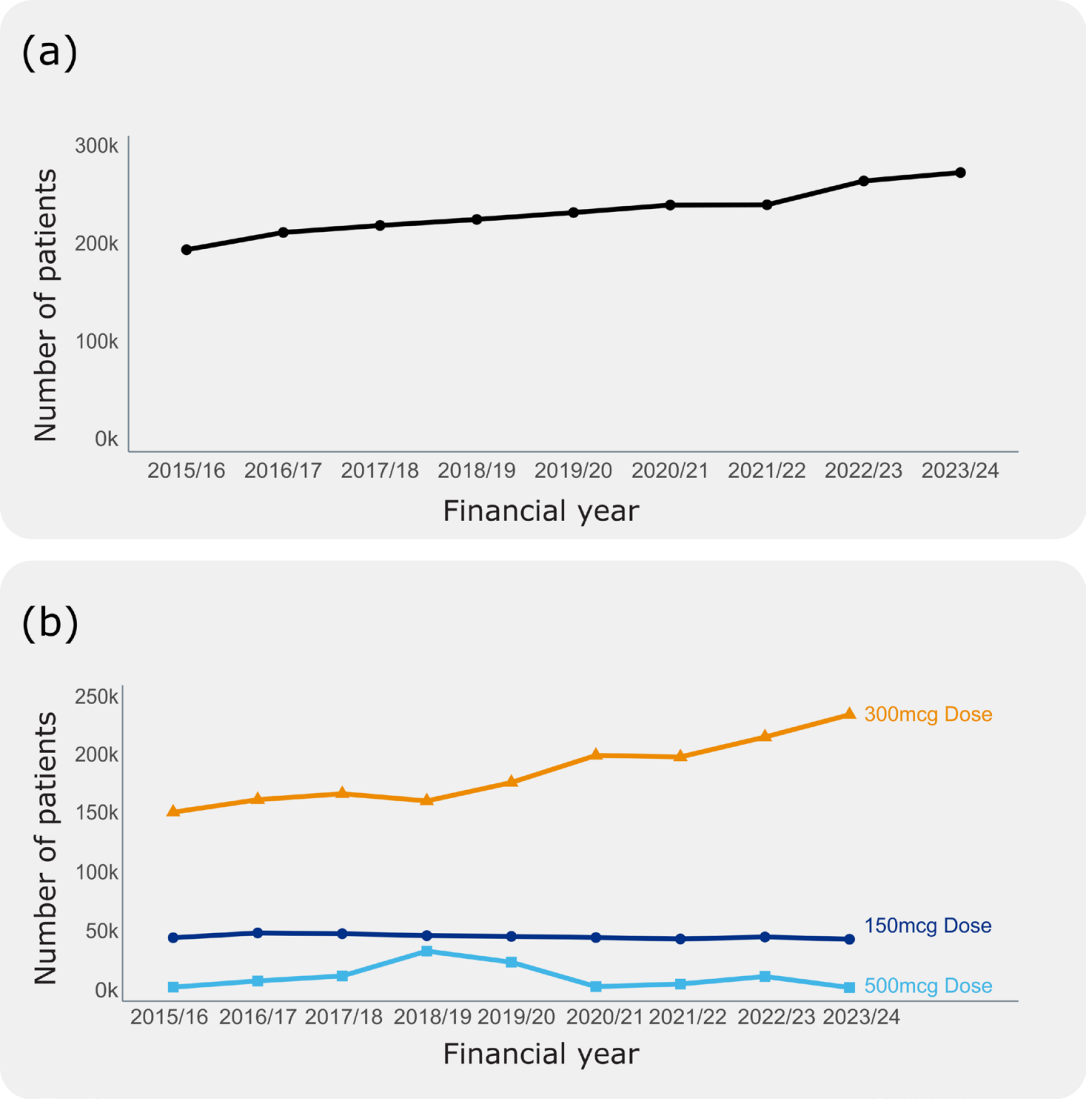
Summary of annual AAI prescribing and dispensing trends across England. (a) A timeseries of prescribing patterns for all Adrenaline Autoinjectors in England between 2015/16 and 2024/23. (b) Prescribing patterns of Adrenaline Autoinjectors have been separated into 150 μg (dark blue), 300 μg (orange) and 500 μg (light blue) auto‐injectors between the 2015/2016 and 2023/2024 financial years.

Nationally, the number of patients receiving a prescription containing an AAI has increased every financial year from 2015 to 2024 (Figure 1a). There was a 41.3% increase in patients receiving an AAI prescription between the financial years of 2015/2016, 193 k patients, and 2023/2024, 277 k patients. 300 μg auto‐injectors are prescribed most often (Figure 1b), followed by 150 μg and 500 μg auto‐injectors, respectively. The 500 μg AAI devices were included for completeness despite being withdrawn from the market in May 2023 [29].

### 3.2 Identification of Patients Who Received a Non‐Standard Dose of Adrenaline

In total, 46,999 patients were identified to have received a 150 μg auto‐injector in their most recent AAI prescription between December 2022 and December 2024.

**FIGURE 2:**
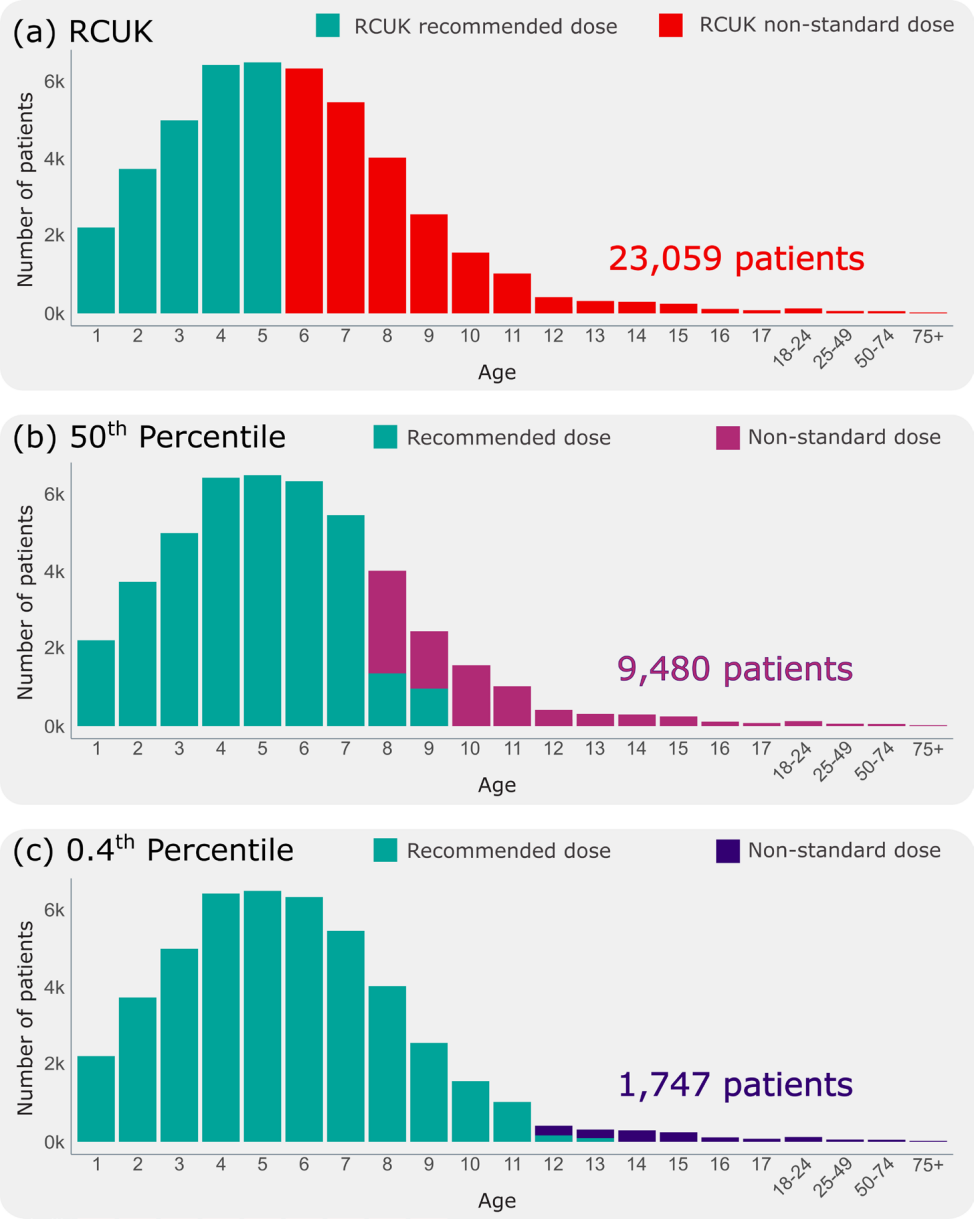
Age distribution of patients whose most recent AAI was a 150 μg auto‐injector between December 2022 and December 2024. (a) For 23,940 (50.9%) patients the prescription of 150 μg auto‐injectors occurred within Resuscitation Council UK (RCUK) guidelines in children under 6 years of age (green). 23,059 (49.1%) patients were identified to have received a 150 μg auto‐injector outside of RCUK guidelines which recommends they should receive a stronger dose auto‐injector (red). (b) For 37,519 (79.8%) patients the prescription of 150 μg auto‐injectors is as expected, occurring in children between 0 and 7 years old (green). 9480 (20.2%) patients were prescribed a 150 μg auto‐injector, but their age suggests that they may benefit from a stronger dose (pink). (c) For 45,252 (96.3%) patients the prescription of 150 μg auto‐injectors is as expected, occurring in children between 0 and 11 years old (green). 1747 (3.7%) patients were prescribed a 150 μg auto‐injector, but their age suggests that they may benefit from a stronger dose (purple).

Using RCUK guidance for switching age, we identified 23,059 patients who received a non‐standard dose (Figure 2a). Using the 50th percentile increases the age at which patients exceed the 25 kg and 30 kg weight thresholds compared to the RCUK recommendation (Figure 2b). As such, the number of patients identified with non‐standard 150 μg auto‐injectors decreases to 9480 (20.2%). An overlap between recommended dose and non‐standard dosing occurs in ages 8 and 9. This occurs as the specific brand of device a patient received is accounted for in this analysis. Patients who received a 150 μg device with a maximum recommended weight of 25 kg should switch by age 8. However, patients who received a device with a 30 kg maximum weight recommendation should switch by age 9.

Using the 0.4th percentile, the prescribing of 150 μg auto‐injectors for 45,252 (96.3%) of patients was believed to be in line with the recommended switching weight (Figure 2c). Using our most conservative estimates of age as a proxy for weight, we identified 1747 (3.7%) patients who have been prescribed a 150 μg auto‐injector but may benefit from a stronger dose based on their age and the brand dispensed. This is not isolated to teenagers close to the expected switching age. Prescriptions of 150 μg auto‐injectors are present for adults of all ages. Similar to the results seen in the 50th percentile, an overlap between recommended dose and non‐standard dosing occurs, now in ages 12 and 13. Again, this occurs as the specific brand of device a patient received is accounted for in this analysis. Results for other percentiles are provided in Figure S1.

We conducted a complementary analysis of non‐standard 300 μg prescriptions, examining how many individuals below the 25–30 kg weight threshold received 300 μg AAIs. Using the most conservative estimate of age (the 99.6th percentile) as a proxy, we assessed national‐level data. For the 25 kg threshold (children under 4 years old), only 67 children receive a non‐standard 300 μg dose. For the 30 kg threshold (children under 5 years old), this number rises to 330.

### 3.3 Number of 150 μg Auto‐Injectors Prescribed to Patients in Their Latest AAI Prescription Across England

**FIGURE 3:**
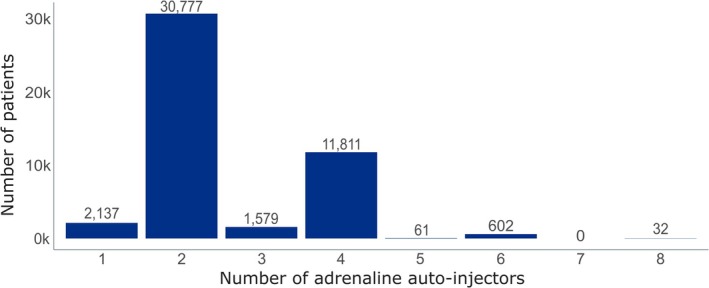
Number of 150 μg auto‐injectors dispensed to patients in their latest Adrenaline Autoinjector prescription. The figure illustrates how many 150 μg auto‐injectors were prescribed and dispensed to patients. Over 65% of prescriptions were for two adrenaline auto‐injector pens. The exact number of prescriptions is provided in the data label above each bar.

NICE guidance on anaphylaxis recommends that patients should be prescribed 2 devices to always carry with them [5]. Looking at patients' most recently dispensed AAI prescription, 150 μg AAIs were typically dispensed as a pair of devices (Figure 3). Over 30,000 (65%) patients received two AAIs. The next most common prescription quantity was for four devices, given to over 11,000 (25%) patients. There were 2137 (4.5%) patients who only received a single AAI in their latest prescription.

### 3.4 Geographic Breakdown of 150 μg Auto‐Injector Prescribing

**FIGURE 4:**
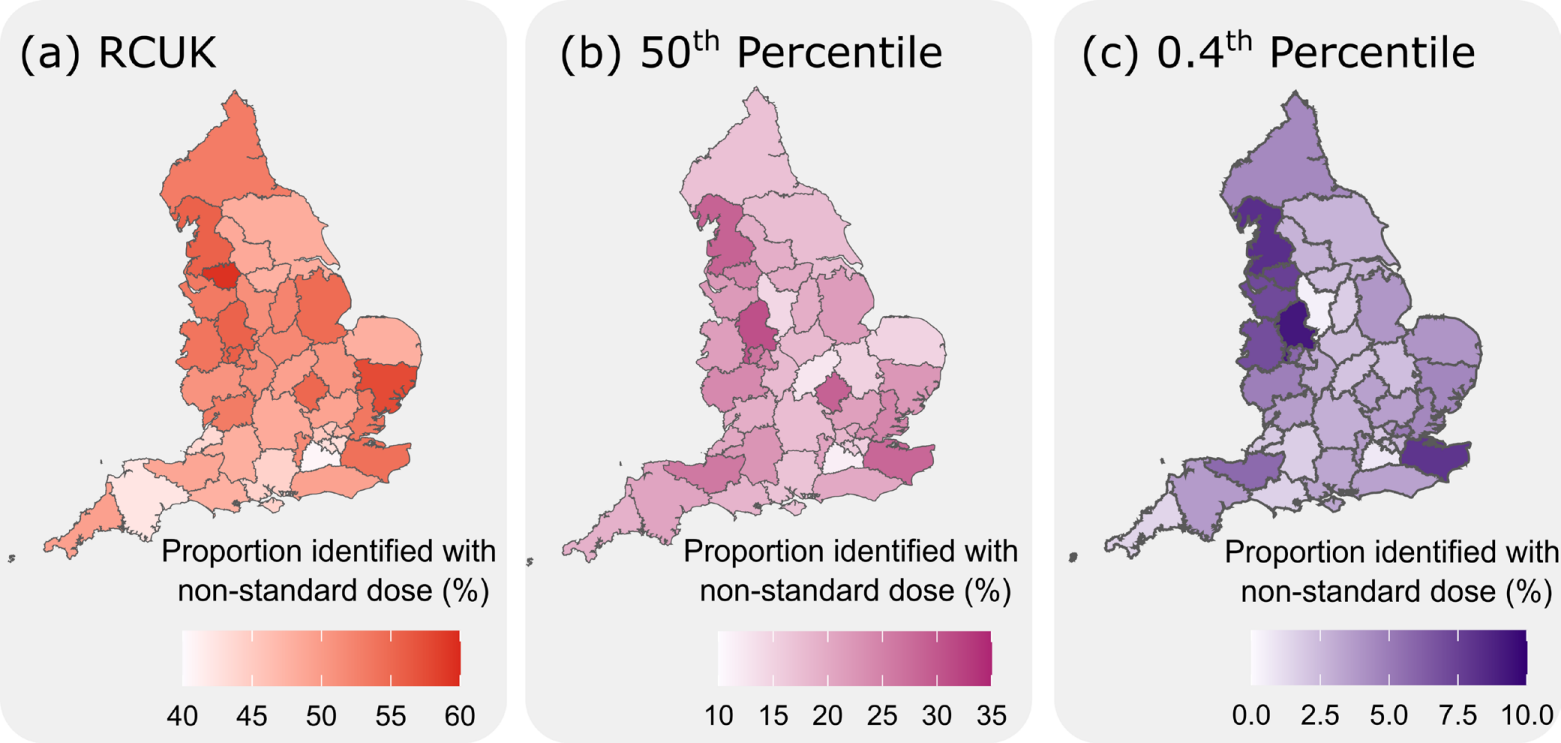
Geographic summary of 150 μg auto‐injector prescribing at an Integrated Care System level using the RCUK, 50th and 0.4th percentile data. (a) The proportion of patients within each ICS who received a 150 μg auto‐injector but may benefit a higher dose based on the switching threshold determined by using the RCUK recommended switching age (a; red), the 50th percentile (b; pink) and 0.4th percentile (c; purple). Darker hues correspond to a higher rate of patients who received a non‐standard dose. The rates of non‐standard prescribing range from the minimum to maximum for each threshold and are not directly comparable across charts.

The proportion of patients who received a 150 μg AAI prescription but may be at risk of requiring a stronger dose, relative to the total number of patients who received a 150 μg prescription within an ICS, was computed. A geographic breakdown illustrates disparities between the ICSs when following the RCUK switching guidelines (Figure 4a), the switching age determined using the 50th percentile (Figure 4b), and the switching age determined using the 0.4th percentile (Figure 4c). Only patients with a known geography were included in the figure (excluding 1761 (3.7%) of patients).

At an ICS level, the proportion of patients who received a non‐standard dose auto‐injector range from 40.57% (NHS Surrey Heartlands) to 59.47% (NHS Greater Manchester) when considering the RCUK switching guidelines, from 11.62% (NHS Surrey Heartlands) to 30.86% (NHS Staffordshire and Stoke‐on‐Trent) when considering the 50th percentile, and from 0.43% (NHS Derby and Derbyshire) to 9.26% (NHS Staffordshire and Stoke‐on‐Trent) when considering the 0.4th percentile. A tabular version of the geographic data for the 0.4th percentile is supplied Table S1. A comparison between the geographic breakdown using the 0.4th and 50th percentiles is provided in Figure S3. A strong significant correlation (Spearman rho = 0.68, *p* < 0.001) exists between the proportion of non‐standard 150 μg doses identified within each ICS when using the 0.4th and 50th percentile age thresholds.

### 3.5 Association Between Deprivation Levels and Proportion of Patients Who Received a Non‐Standard Dose AAI

**FIGURE 5:**
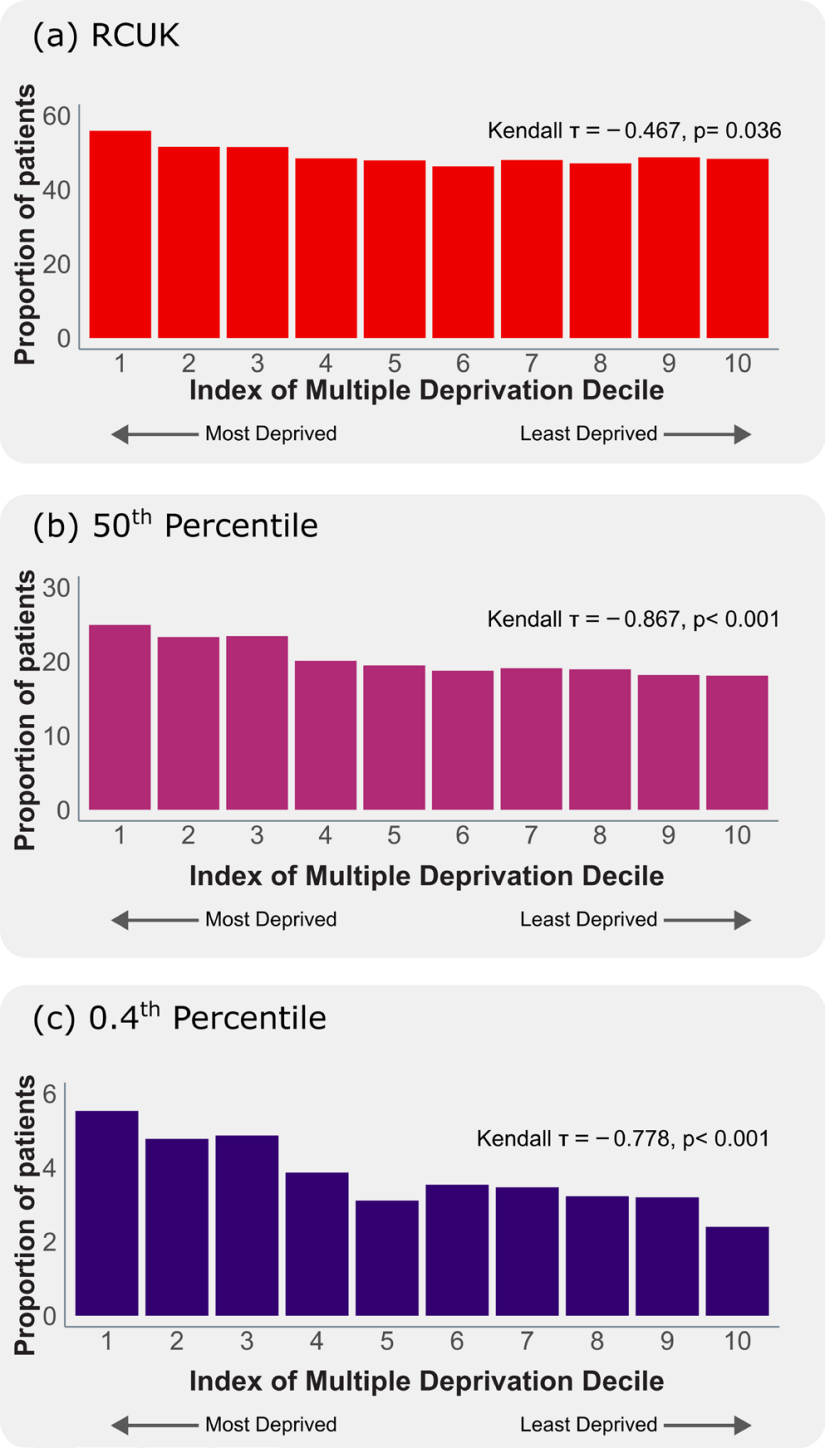
Association between the proportion of patients that may benefit from a stronger dose of AAI based on the RCUK, 50th percentile and the 0.4th percentile thresholds and the Index of Multiple Deprivation Decile. Each bar corresponds to the average proportion of patients who received a non‐standard dose of AAI within each IMD decile. Each decile corresponds to 10% of LSOAs. For example, the 1st decile corresponds to the 10% of LSOAs that are most deprived and the 10th decile the 10% of LSOAs that are least deprived. A negative correlation was observed, quantified by a statistically significant Kendall's tau correlation coefficient. Only patients with a known geography were included (this excludes 1761 (3.7%) of patients).

The rate of patients who received a non‐standard dose AAI and the IMD deciles were compared using Kendall's tau correlation. The rate of non‐standard AAI prescribing was higher in areas with a greater index of multiple deprivation (Figure 5). This was the case for all three methods used for estimating age at transition to a 300 μg AAI dose. A comparison between ICS deprivation rank (in place of decile) and the rates of non‐standard 150 μg prescribing can be seen in Figure S2 and highlights a similar correlation, with a lower rate of non‐standard prescribing in less deprived areas.

## 4 Discussion

This novel study of national level Primary Care NHS prescriptions data highlights inconsistencies in AAI dose prescribing. We demonstrated using the 0.4th percentile, our most conservative estimate of when patients reach the recommended switching weight of 25–30 kg, that (1747) 3.7% of patients receive non‐standard AAI doses across the whole of England. That is, they received a 150 μg dose AAI when their age suggests a 300 μg dose may be more appropriate. Using RCUK guidelines, which recommend switching based on age alone and not weight, we found that (20,059) 49.1% of patients received a non‐standard dose. Our analysis using the 50th percentile provides a balance between our very conservative 0.4th percentile analysis and the RCUK guidelines. From our analysis, we show that over 99.6% of the population exceed the 25 kg and 30 kg thresholds at age 12 and 14, respectively. On this basis, we suggest that automated safety procedures are put in place to identify these individuals that may be on a non‐standard 150 μg dose. We acknowledge that most patients will exceed the 25 kg and 30 kg threshold sooner than ages 12 and 14 as evident from the analysis using the 50th percentile, where 50% of the population exceed 25 kg and 30 kg at ages 8 and 10.

Between 2015/2016 and 2023/2024, patients receiving AAI prescriptions increased by 41.3% in England (Figure 1). This is likely due to greater awareness and education on allergic diseases [30, 31] and workforce up‐skilling [32]. The rise in 300 μg AAI prescriptions suggests either a potentially growing disease prevalence—though recent studies challenge this [33]—or better recognition by professionals [15] and educators [34].

Using the 50th and 0.4th percentile to estimate switching age, our study showed that, for 37,519 (79.2%) and 45,252 (96.3%) of patients, respectively, the prescribing of 150 μg AAIs was believed to be in line with the recommended switching weight (Figure 2b,c). However, based on their age and the brand dispensed, there were 9480 (20.2%) and 1747 (3.7%) patients, respectively, identified who have been prescribed a non‐standard 150 μg AAI. These patients may therefore benefit from a stronger dose. It is important to note that the data capture period for our study was after the most recent RCUK guideline update in August 2021. The majority of the 1747 patients in receipt of a non‐standard dose AAI when considering the most conservative 0.4th percentile were young adults (12–22 years of age). This age group is at the highest risk of anaphylaxis, and more attention is required relating to patient awareness, education, prescription and treatment [35, 36, 37]. Like other chronic diseases, a well‐structured transition of care from childhood to adulthood would significantly benefit patients living with allergies and asthma. A national drive continues to enhance services and transition processes for young adults across all healthcare settings [35, 37, 38]. This is not isolated to teenagers close to the expected switching age but includes adults of all ages. Whilst this might be a shortfall on behalf of professionals prescribing the AAI, it is known that as the body ages, changes can decrease the body's ability to metabolise certain medications. Older adults may need a lower dose of the medication or a different medication that is safer due to side effects common in the geriatric group [39, 40]. Alternatively, older adults are more likely to be co‐prescribed beta blockers, which counter the effect of AAI devices [41]. This further emphasises the need for prescribing the correct AAI dose and appropriate regular prescribing reviews.

Following the RCUK guidance [7] that children should be switched to a 300 μg dose AAIs at age 6, identified 23,059 patients received a non‐standard dose AAI. Given the disparities observed using the RCUK recommendation, 50th percentile and 0.4th percentile data (Figure 2), we suggest primary care prescribing guidance must be reviewed to include updated recommendations around switching patients from 150 μg to 300 μg devices. One approach to guide prescribers may be to use the estimated switching ages of 8 and 10 years for the 25 kg and 30 kg thresholds, respectively, based on the 50th percentile of the RCPCH growth charts. This is where half the population at that age are expected to exceed the weights. Therefore, patients dispensed EpiPen 150 μg, with a switching weight of 25 kg should be reviewed at age 8, whereas those dispensed Jext or Emerade 150 μg, with a switching weight of 30 kg should be reviewed at age 10. This is key as there are often discrepancies between patient weights and the recommended AAI dose depending on the brand [42].

Our data showed that over 95% of patients received 2 or more AAI devices (Figure 3), as per recommendations [5]. Prescribing multiple devices for children is to be expected, as most families can be prescribed more than one set of devices to ensure adequate access in schools [43, 44]. It has also been highlighted that, on occasion, more than two AAIs may be required for the resolution of fatal anaphylaxis episodes [45]. Together, these two factors may contribute to the tensions in AAI availability. Although we identified patients who received a single AAI, we cannot know if this means that they were prescribed against recommendations or if they were replacing a single device because a previous pair of AAIs had differing expiry dates. Although it has been well described for almost a decade that there is an insufficient carriage of AAI devices among individuals who are at risk of anaphylaxis [45, 46, 47]. It should be noted that receipt of two 150 μg devices does not equate to a 300 μg device. While it is encouraging that most prescribers adhere to guidelines regarding the quantity of AAIs dispensed, greater emphasis is needed on assessing patient age and weight to ensure appropriate dosing.

To reduce the financial burden of AAI prescribing, it has been proposed that education providers are supplied with generic AAI devices, thus mitigating the need for children and young people needing multiple sets of devices. The Spare Pen in School initiative highlights the vital importance of having generic AAIs available for use in cases of anaphylaxis in school settings [48] to be consistent with standard care. If this programme were to be made a statutory requirement for all schools then each pupil would only require prescription of a single AAI for the learning environment instead of two devices. The potential saving to the NHS in England is currently being evaluated during pilot work in schools by the Beat Anaphylaxis Group [49, 50]. Our study also highlights the need for increased patient and healthcare professional awareness and education to ensure patients receive the appropriate number of devices at the correct dose, with a focus on young adults where no elicited trigger is identified [45].

When looking at associations between rates of non‐standard prescribing and demographic factors such as geography and deprivation, we continued to focus on the estimates using the RCUK, 50th percentile, and 0.4th percentile thresholds. The rationale for the 0.4th percentile is so that we are as confident as we can be that the population is indeed those with non‐standard dose AAIs. Compared to the 50th percentile, this may not be as reflective of the true population of patients with non‐standard dose AAIs, but it is more accurate in terms of only identifying those who have exceeded the 25 kg and 30 kg weight thresholds for 150 μg AAI prescribing. Regardless of how non‐standard dosing is defined (using RCUK (Figure 4a), 50th percentile (Figure 4b) and 0.4th percentile (Figure 4c)), there is a lot of variation across England. For RCUK, rates range from approximately 40% to 60%, the 50th percentile from 10%–30%, and 0%–10% for the 0.4th percentile. Across different ICS areas, the patterns of non‐standard prescribing are quite similar whether you look at the 0.4th percentile or the 50th percentile (Spearman rho = 0.68, *p* < 0.001 (Figure S2)). In other words, if an area has high rates at one level, it usually has high rates at the other too.

The proportion of patients who received a non‐standard dose AAI varied with deprivation. Statistical comparison between rates of non‐standard prescribing using the RCUK, 50th and 0.4th percentile thresholds and IMD decile demonstrates a statistically significant negative association (Figure 5). This is consistent with other domains of allergy care, which suggest that areas with higher deprivation are associated with a larger proportion of patients either unable or not accessing care [24].

## 5 Recommendations

The BSACI guidance for primary care on AAI prescribing for patients at risk of anaphylaxis highlights (i) how to identify patients at high risk of anaphylaxis, (ii) the need for referral to a specialist allergy clinic, (iii) the importance of an allergy‐focused history, (iv) appropriate investigations, and (v) the need to have two AAIs for those at significant risk of anaphylaxis as per Medicine and Healthcare Regulatory Agency report (MHRA).

The findings in this analysis indicate that rates of non‐standard prescribing differ across England. We suggest that each ICS conduct a patient safety review to identify patients within their area who are in receipt of a non‐standard AAI dose.

Using the 50th percentile of the RCPCH growth charts gives an age of 8 years old where at least 50% of the population would be expected to exceed the lower weight threshold of 25 kg. We therefore suggest that at 8 years old all patients are reviewed to ensure they are on the appropriate dose AAI for their weight. This could be incorporated into the next update of NICE CG134 [5] guidance. In addition, as a secondary safety net to prevent long‐term underdosing, we suggest that an automated flag is embedded into primary care systems in England to alert healthcare professionals about non‐standard 150 μg prescribing in patients over 14 years old. At this age, more than 99.6% of the population will have exceeded the 30 kg threshold, and therefore prescribing 150 μg will likely result in an underdose. This will reduce the risk of non‐standard treatment of anaphylaxis in those older than 14 years old where the risk of underdose is highest.

There is a growing need to explore the role of UK pharmacists in patient education and the delivery of appropriate adrenaline doses [51, 52]. With the recent approval of neffy epinephrine intranasal spray in the USA and Europe for the treatment of anaphylaxis, staying up to date and educating patients, healthcare professionals and pharmacists is becoming increasingly important [53, 54]. It is important that AAI prescriptions are not considered a substitute for referrals.

Hospital‐based anaphylactic episodes, occurring under medical supervision, should be utilised to ensure each patient is discharged with the appropriate dosage AAI. This approach places Anaphylaxis Prevention at the core of the National Allergy Strategy, driving efforts to reduce allergic diseases and anaphylaxis on a national scale [55].

## 6 Limitations

This analysis is not without limitations. First, NHS prescriptions data lack clinical indication, meaning we know when adrenaline is prescribed and dispensed but not why. However, AAI prescribing is limited to acute anaphylaxis, which minimises this. Furthermore, NHSBSA data do not capture the expiry date of dispensed AAI devices; for this reason, analysis is limited to the latest 2‐year window (at the time of analysis), which corresponds to the maximum shelf life of an AAI device. Second, NHSBSA data cover only primary care prescriptions in England, excluding private and secondary care, though this impact is minimal as NHS primary care is the main route for ongoing AAI prescribing. Finally, our data are limited to England; future studies could extend this analysis to Scotland, Wales, and Northern Ireland for a full UK assessment of non‐standard AAI prescribing.

## 7 Conclusion

This is the first time that data covering all NHS prescriptions dispensed in the community in England has been used to assess anaphylaxis treatment. We use novel techniques and information on the dispensed devices to estimate the approximate patient age when they reach the recommended weight switching thresholds. Our analysis of 150 μg AAI prescribing demonstrates that non‐standard AAI prescribing occurs across the whole of England and across all ages. We recommend that primary care systems are reviewed and automated triggers are introduced to minimise the risk of non‐standard prescribing. In the absence of patient weight, we recommend that professionals use our estimates of age to determine whether patients should be switched to a stronger dose AAI.

## Author Contributions

L.J.M.: conceptualisation, investigation, writing – original draft, supervision, resources, and writing – review and editing. T.O.: conceptualisation, investigation, data curation, formal analysis, writing – original draft, supervision, resources, and writing – review and editing. A.D.B.: conceptualisation, investigation, writing – original draft, resources, and writing – review and editing. L.S.‐R.: conceptualisation, investigation, data curation, formal analysis, writing – original draft, supervision, resources, and writing – review and editing.

## Conflicts of Interest

A.D.B. has no direct conflicts of interest regarding this publication. He is a member of the Ready2React campaign (supported by ALK Viatris), a member of the National Allergy Strategy Group, and is Clinical Lead for the Beat Anaphylaxis Project (supported by ALK and Viatris). L.J.M. has no direct conflicts of interest regarding this publication. She has participated in current (ALK) and previous (Danone, Regeneron) clinical trials. She is a member of the Beat Anaphylaxis Project (supported by ALK and Viatris), the BSACI vice president for services, and is the BSACI Secretary for the Immunotherapies Registry. L.S.‐R. and T.O. have no conflicts of interest to declare.

## Supporting information


**Figure S1:** Age distribution of patients whose most recent AAI was a 150 μg auto‐injector between December 2022 and December 2024.
**Figure S2:** Comparison of ICS rates of non‐standard AAI doses when using the 0.4th percentile and 50th percentile estimates.
**Figure S3:** Comparison of the proportion of patients who received a non‐standard dose and the IMD deprivation rank of the ICS.
**Table S1:** ICS summary of patients who received a non‐standard dose.

## Data Availability

All data used in this study is anonymised and held internally by the NHS Business Services Authority. Appendix S1 contains all the data required to recreate results in this analysis. The underlying data cannot be shared publicly as they contain patient‐level sensitive information.
