# Supplementary material for: Adrenaline Auto‐Injector Prescribing in Primary Care in England: An Analysis of Non‐Standard Dosing
Source: Clin Exp Allergy. 2025 Nov 2;56(1):30–40. doi: 10.1111/cea.70163 (PMC12774575; doi:10.1111/cea.70163)
Supplement: Supplementary file 1 — Figure S1: Age distribution of patients whose most recent AAI was a 150 μg auto‐injector between December 2022 and December 2024. Figure S2: Comparison of ICS rates of non‐standard AAI doses when using the 0.4th percentile and 50th percentile estimates. Figure S3: Comparison of the proportion of patients who received a non‐standard dose and the IMD deprivation rank of the ICS. Table S1: ICS summary of patients who received a non‐standard dose. [file CEA-56-30-s001.docx]

**Adrenaline auto-injectors in England: Non-standard 150mcg dosing in primary care supplement**

Louise J Michaelis MD PhD^1,2^, Thomas Owen PhD^3^, Andrew D Bright MD^1,4^, Lucy Sherwin-Robson PhD^3^

^1^ Newcastle Upon Tyne Hospitals NHS Foundation Trust, Newcastle Upon Tyne, NE1 4LP

^2^ Population Health Sciences Institute, Newcastle University, Newcastle Upon Tyne, NE2 4HH

^3^ NHS Business Services Authority

^4^ Gateshead Health NHS Foundation Trust, Queen Elizabeth Hospital, NE9 6SX

**Corresponding author**

Louise J Michaelis

Newcastle Upon Tyne Hospitals NHS Foundation Trust, Newcastle Upon Tyne, NE1 4LP

Population Health Sciences Institute, Newcastle University, Newcastle Upon Tyne, NE2 4HH

[Louise.michaelis@nhs.net](mailto:Louise.michaelis@nhs.net)

Table of Contents

[Supplementary Tables 2](#_Toc206680157)

[Table S1: ICS summary of patients who received a non-standard dose. 2](#_Toc206680158)

[Supplementary Figures 3](#_Toc206680159)

[Figure S1: Age distribution of patients whose most recent AAI was a 150mcg auto-injector between December 2022 and December 2024. 4](#_Toc206680160)

[Figure S2: Comparison of ICS rates of non-standard AAI doses when using the 0.4th percentile and 50th percentile estimates. 5](#_Toc206680161)

[Figure S3: Comparison of the proportion of patients who received a non-standard dose and the IMD deprivation rank of the ICS 6](#_Toc206680162)

[Supplementary Methods 7](#_Toc206680163)

[Selecting relevant AAI prescriptions 7](#_Toc206680164)

[NHSBSA prescription data caveats 7](#_Toc206680165)

## Supplementary Tables

### Table S1: ICS summary of patients who received a non-standard dose.

| **ICS Name** | **Total Number of patients whose latest AAI prescription is 150mcg** | **Number of patients in receipt of a non-standard dose** | **Proportion of patients in receipt of a non-standard dose (%)** | **ICS Deprivation Rank** |
| --- | --- | --- | --- | --- |
| NHS Bath and North East Somerset, Swindon and Wiltshire ICS | 348 | 6 | 1.72 | 38 |
| NHS Bedfordshire, Luton and Milton Keynes ICS | 998 | 35 | 3.51 | 30 |
| NHS Birmingham and Solihull ICS | 1483 | 53 | 3.57 | 1 |
| NHS Black Country ICS | 1029 | 61 | 5.93 | 2 |
| NHS Bristol, North Somerset and South Gloucestershire ICS | 468 | 9 | 1.92 | 23 |
| NHS Buckinghamshire, Oxfordshire and Berkshire West ICS | 1838 | 54 | 2.94 | 41 |
| NHS Cambridgeshire and Peterborough ICS | 644 | 15 | 2.33 | 35 |
| NHS Cheshire and Merseyside ICS | 993 | 71 | 7.15 | 6 |
| NHS Cornwall and the Isles of Scilly ICS | * | * | 1.44 | 11 |
| NHS Coventry and Warwickshire ICS | 828 | 26 | 3.14 | 24 |
| NHS Derby and Derbyshire ICS | * | * | 0.43 | 18 |
| NHS Devon ICS | 748 | 28 | 3.74 | 21 |
| NHS Dorset ICS | 496 | 8 | 1.61 | 34 |
| NHS Frimley ICS | 866 | 32 | 3.70 | 40 |
| NHS Gloucestershire ICS | 220 | 8 | 3.64 | 37 |
| NHS Greater Manchester ICS | 1663 | 126 | 7.58 | 3 |
| NHS Hampshire and Isle of Wight ICS | 1519 | 50 | 3.29 | 31 |
| NHS Herefordshire and Worcestershire ICS | 307 | 15 | 4.89 | 28 |
| NHS Hertfordshire and West Essex ICS | 1988 | 71 | 3.57 | 39 |
| NHS Humber and North Yorkshire ICS | 805 | 22 | 2.73 | 16 |
| NHS Kent and Medway ICS | 1428 | 115 | 8.05 | 20 |
| NHS Lancashire and South Cumbria ICS | 676 | 56 | 8.28 | 8 |
| NHS Leicester, Leicestershire and Rutland ICS | 990 | 24 | 2.42 | 29 |
| NHS Lincolnshire ICS | 358 | 14 | 3.91 | 19 |
| NHS Mid and South Essex ICS | 1480 | 67 | 4.53 | 32 |
| NHS Norfolk and Waveney ICS | 501 | 20 | 3.99 | 14 |
| NHS North Central London ICS | 2101 | 75 | 3.57 | 12 |
| NHS North East and North Cumbria ICS | 1545 | 69 | 4.47 | 7 |
| NHS North East London ICS | 2824 | 144 | 5.10 | 9 |
| NHS North West London ICS | 2665 | 52 | 1.95 | 15 |
| NHS Northamptonshire ICS | 602 | 13 | 2.16 | 26 |
| NHS Nottingham and Nottinghamshire ICS | 690 | 12 | 1.74 | 10 |
| NHS Shropshire, Telford and Wrekin ICS | 158 | 11 | 6.96 | 22 |
| NHS Somerset ICS | 260 | 15 | 5.77 | 27 |
| NHS South East London ICS | 2679 | 74 | 2.76 | 13 |
| NHS South West London ICS | 2281 | 40 | 1.75 | 36 |
| NHS South Yorkshire ICS | 674 | 16 | 2.37 | 4 |
| NHS Staffordshire and Stoke-on-Trent ICS | 486 | 45 | 9.26 | 17 |
| NHS Suffolk and North East Essex ICS | 656 | 30 | 4.57 | 25 |
| NHS Surrey Heartlands ICS | 1427 | 10 | 0.70 | 42 |
| NHS Sussex ICS | 1189 | 41 | 3.45 | 33 |
| NHS West Yorkshire ICS | 1586 | 44 | 2.77 | 5 |
| Unknown | 1761 | 64 | 3.63 | N/A |

The table is ordered from highest to lowest based on the proportion of patients who received a 150mcg auto-injector but may need a stronger dose. The ICS deprivation rank ranges from 1 to 42 where 1 corresponds to the most deprived ICS and 42 the least deprived. In line with the National Health Service Business Services Authority's Statistical Disclosure Control Policy, some data has been excluded (marked with an *) where if included there may have been a potential risk of patient identification.

## Supplementary Figures

### Figure S1: Age distribution of patients whose most recent AAI was a 150mcg auto-injector between December 2022 and December 2024.


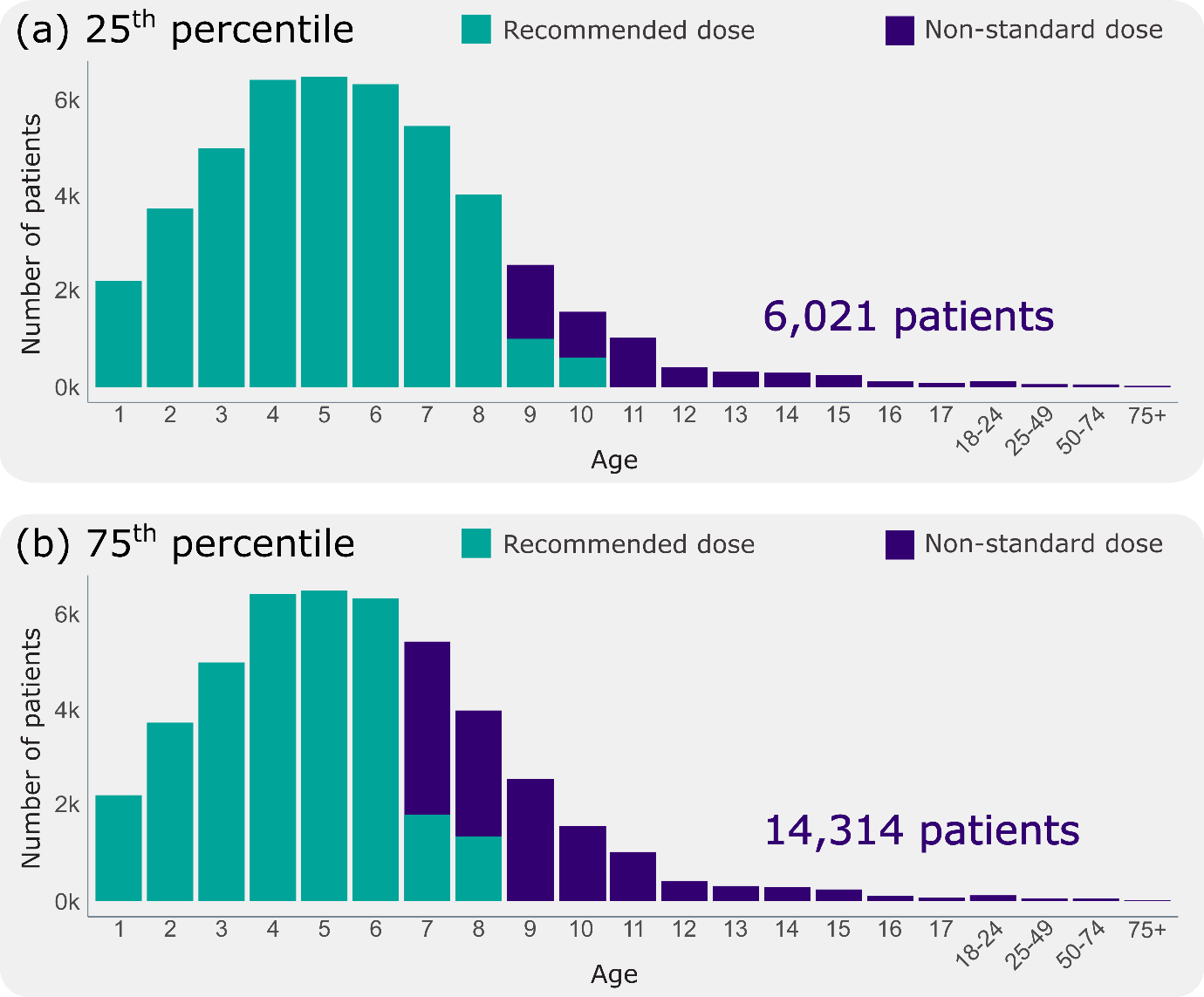


**(a)** Using the 25th percentile 40,978 (87.2%) patients received a 150mcg auto-injector prescription as expected, occurring in children between 0-10 years old (green). 6,021 (12.8%) patients were prescribed a 150mcg auto-injector, but their age suggests that they may benefit from a stronger dose (purple). **(b)** Using the 75th percentile 32,685 (69.5%) patients received a 150mcg auto-injector prescription as expected, occurring in children between 0-8 years old (green). 14,314 (30.5%) patients were prescribed a 150mcg auto-injector, but their age suggests that they may benefit from a stronger dose (purple).

### Figure S2: Comparison of ICS rates of non-standard AAI doses when using the 0.4th percentile and 50th percentile estimates.


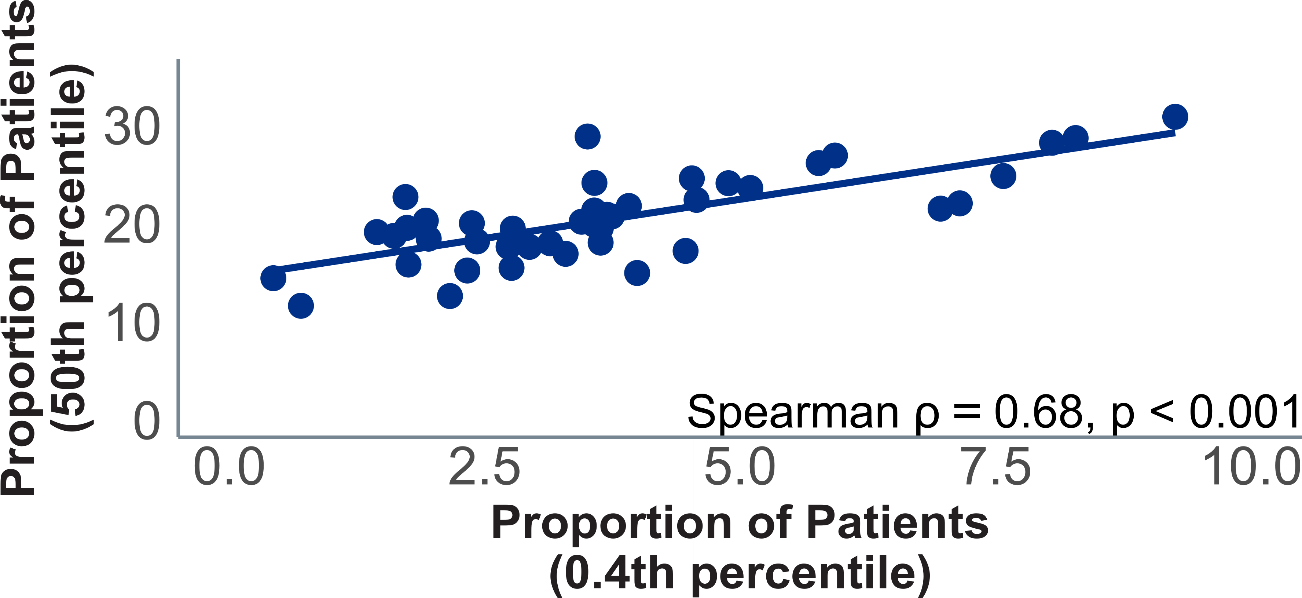


Scatter plot depicts the relationship between non-standard 150mcg AAI prescribing when the switching age has been identified using the 0.4^th^ and 50^th^ percentile. Each point corresponds to a single ICS. A strong and statistically significant association is observed, with a spearman correlation of 0.68.

### Figure S3: Comparison of the proportion of patients who received a non-standard dose and the IMD deprivation rank of the ICS


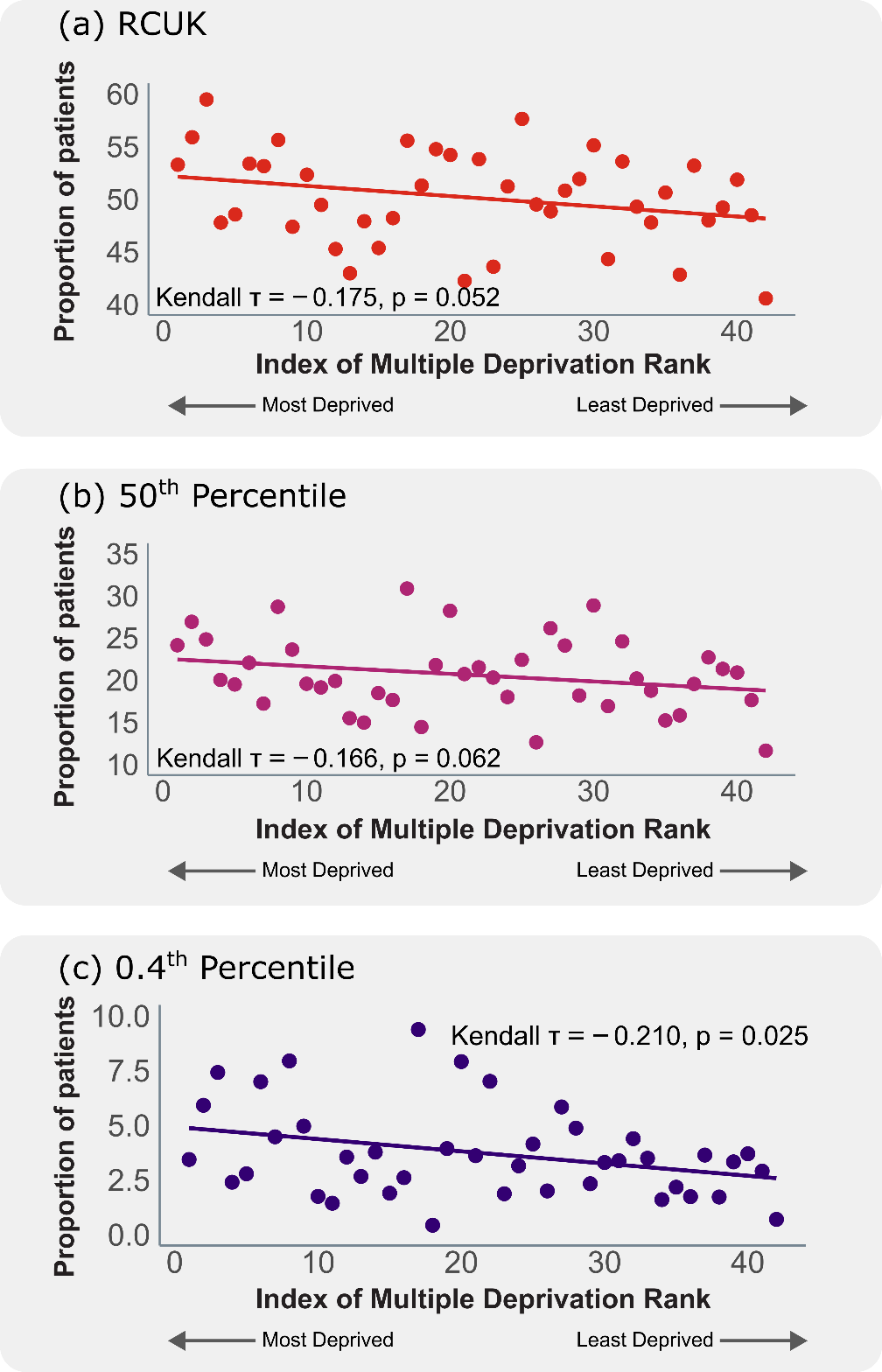


The scatter plots illustrate the relationship between deprivation and the proportion of patients who received a non-standard dose 150mcg auto-injector based on the switching threshold determined by using the RCUK recommended switching age (a; red), the 50th percentile (b; pink) and 0.4th percentile (c; purple). Each point corresponds to an ICS. ICSs in the most deprived regions have lower IMD ranks. The relationship between the two features and its level of statistical significance was calculated using a one-tailed Kendall’s correlation test.

## Supplementary Methods

### Selecting relevant AAI prescriptions

Analysis in this report is based on electronic and paper-based NHS prescriptions dispensed in the community in England. This does not include medicines used in secondary care, prisons, issued by a private prescriber, or over-the-counter. It does not contain any personally identifiable information.

Prescriptions were included in this analysis if they were dispensed a 150mcg AAI within the last 24 months. At the time of data processing this was prescriptions dispensed between December 2022 and December 2024, this ensures expired prescriptions are excluded.

For patients with multiple AAI prescriptions between December 2022 and December 2024 we retained only the most recent prescription.

Prescriptions of 150mcg AAI’s prescribed and/or dispensed generically (as a virtual medicinal product (VMP)) were assigned to the only current actual medicinal product (AMP) for that VMP so that the appropriate switching threshold for patient weight could be ascertained. The assignments for generically prescribed products were based on the only current valid AMP listed under the corresponding VMP in the dictionary of medicines and devices, and are as follows:

- - Adrenaline (base) 150mcg/0.3ml (1in2,000) inj pf dispos dev (0304030C0AAA2A2) were assigned to EpiPen Jr. 150microg/0.3ml (1 in 2,000) inj auto-injectors (0304030C0BEAAA2)
  - Adrenaline (base) 150mcg/0.15ml (1in1,000) inj pf dispos dev (0304030C0AABFBF) were assigned to Jext 150micrograms/0.15ml (1 in 1,000) inj auto-injectors (0304030C0BHAABF). .

### NHSBSA prescription data caveats

Below are the standard data caveats and filters applied to the prescription data.

#### NHSBSA data exclusions:

The NHSBSA:

- Excludes prescriptions that were issued but not presented for dispensing.
- Excludes prescriptions that were not submitted to the NHSBSA for processing and reimbursement.
- Excludes prescriptions issued and dispensed in prisons, hospitals and private prescriptions.
- Excludes items not dispensed, disallowed and those returned to the contractor for further clarification.
- Limits to prescription items both prescribed and dispensed in England.
- Prescription data relates to prescription batches submitted to the NHSBSA for payment between April 2015 and March 2024, although most analysis is limited to prescriptions dispensed between December 2022 and December 2024. The month in NHSBSA data relates to the dispensing month for which the prescription batch was submitted. This is generally, but not always, the month in which the prescription was dispensed. This means that there may be dispensing that has not been submitted to the NHSBSA for payment and is therefore not included. There may also be prescriptions included that were dispensed prior to the dispensing month.
- Where analysis is based on patient information this is limited to prescribing where the patient can be identified from the prescription data. NHS numbers are captured for 100% electronic prescription messages. The estimated NHS number capture rate for paper prescription forms is 83.7%.
- All analysis based on geographical location, including IMD deciles, are limited to prescribing for patients identified with English residential addresses.

#### Data Classifications (General):

- Due to manual processes involved in the processing of prescriptions there may be inaccuracies in capturing prescription information which are then reflected in the data. NHS Prescription Services have a variety of validation streams throughout prescription processing to support accurate capture of the data. In addition, a retrospective sample is completed in the month following reimbursement to identify the accuracy of prescription processing information. The check includes the accuracy of prescriber, practice and drug information, but does not include the personal details of the patient. The latest reported Prescription Processing Information Accuracy is 99.9%, which covers the 12-month rolling period ending April 2024. The sample may not be representative at a more granular level; as such the level of accuracy is undetermined for specific groups such as drugs, geographies, time periods etc. It should also be noted that the identification of errors in the accuracy checking sample does not result in amendments to data held in NHSBSA systems. Further Prescription Processing Information Accuracy can be found here.

#### Data Classifications (Patient):

- The NHSBSA periodically investigate the accuracy of NHS numbers captured from paper forms. The personal details captured (NHS number, date of birth and age) are compared against those on the prescription form for a random sample of 50,000 prescription forms. The NHS number captured typically matches that on the prescription form for over 99.9% of forms. The results represent the accuracy for all items processed; as such the level of accuracy is undetermined for specific medications, geographies, time periods and other factors. By contrast, the accuracy of captured NHS numbers in electronic prescribing is estimated to be 100%.
- Patient age was determined using a mixture of patient information from prescription forms and Personal Demographic Service (PDS), based on logic determined by NHSBSA Data Warehouse Team.
  - The Personal Demographic Service (PDS) is a part of NHS England that holds information that allows healthcare professionals to identify patients and match them to their health records. This includes information such as NHS number, date of birth, gender, registered address, and registered GP practice.
  - Each month when data is loaded into the NHSBSA Data & Insight Data Warehouse, NHS numbers that have been captured are sent to PDS to verify them. That list includes all NHS numbers that were scanned in that month and previously verified NHS numbers that have a birthday in that month. Details held by PDS are returned to the NHSBSA, including updates to previously verified NHS numbers.
  - Because this process takes time, new and updated verified data from PDS is loaded into the NHSBSA Data & Insight Data Warehouse the month after the NHS numbers were first scanned. For example, a new NHS number received in January and subsequently verified would be classed as ‘not verified’ in January and ‘verified’ in February. In February the additional information about that patient such as gender and age would become available.
- The NHSBSA does not capture information relating to a patient’s sex or gender from a prescription during processing activities. Gender is instead obtained from PDS, where a patient can be matched based on the captured NHS number. The latest available gender classification was used for each patient, as available at the time of publication. For this report data was limited to patients identified as female via the PDS dataset.
- Geographic reporting is based on the patient's residential address on their latest prescription for relevant prescribing activity, as captured from either the prescription data or identified from data reported by PDS.
